# Getting the chronological age out of DNA: using insights of age-dependent DNA methylation for forensic DNA applications

**DOI:** 10.1007/s13258-023-01392-8

**Published:** 2023-05-30

**Authors:** Jana Naue

**Affiliations:** Institute of Forensic Medicine, Medical Center-University of Freiburg, Faculty of Medicine, University of Freiburg, Freiburg, Germany

**Keywords:** Forensic epigenetics, DNA methylation, Chronological age prediction, Age-dependent changes

## Abstract

**Background:**

DNA analysis for forensic investigations has a long tradition with important developments and optimizations since its first application. Traditionally, short tandem repeats analysis has been the most powerful method for the identification of individuals. However, in addition, epigenetic changes, i.e., DNA methylation, came into focus of forensic DNA research. Chronological age prediction is one promising application to allow for narrowing the pool of possible individuals who caused a trace, as well as to support the identification of unknown bodies and for age verification of living individuals.

**Objective:**

This review aims to provide an overview of the current knowledge, possibilities, and (current) limitations about DNA methylation-based chronological age prediction with emphasis on forensic application.

**Methods:**

The development, implementation and application of age prediction tools requires a deep understanding about the biological background, the analysis methods, the age-dependent DNA methylation markers, as well as the mathematical models for age prediction and their evaluation. Furthermore, additional influences can have an impact. Therefore, the literature was evaluated in respect to these diverse topics.

**Conclusion:**

The numerous research efforts in recent years have led to a rapid change in our understanding of the application of DNA methylation for chronological age prediction, which is now on the way to implementation and validation. Knowledge of the various aspects leads to a better understanding and allows a more informed interpretation of DNAm quantification results, as well as the obtained results by the age prediction tools.

## Introduction

**Fig. 1:**
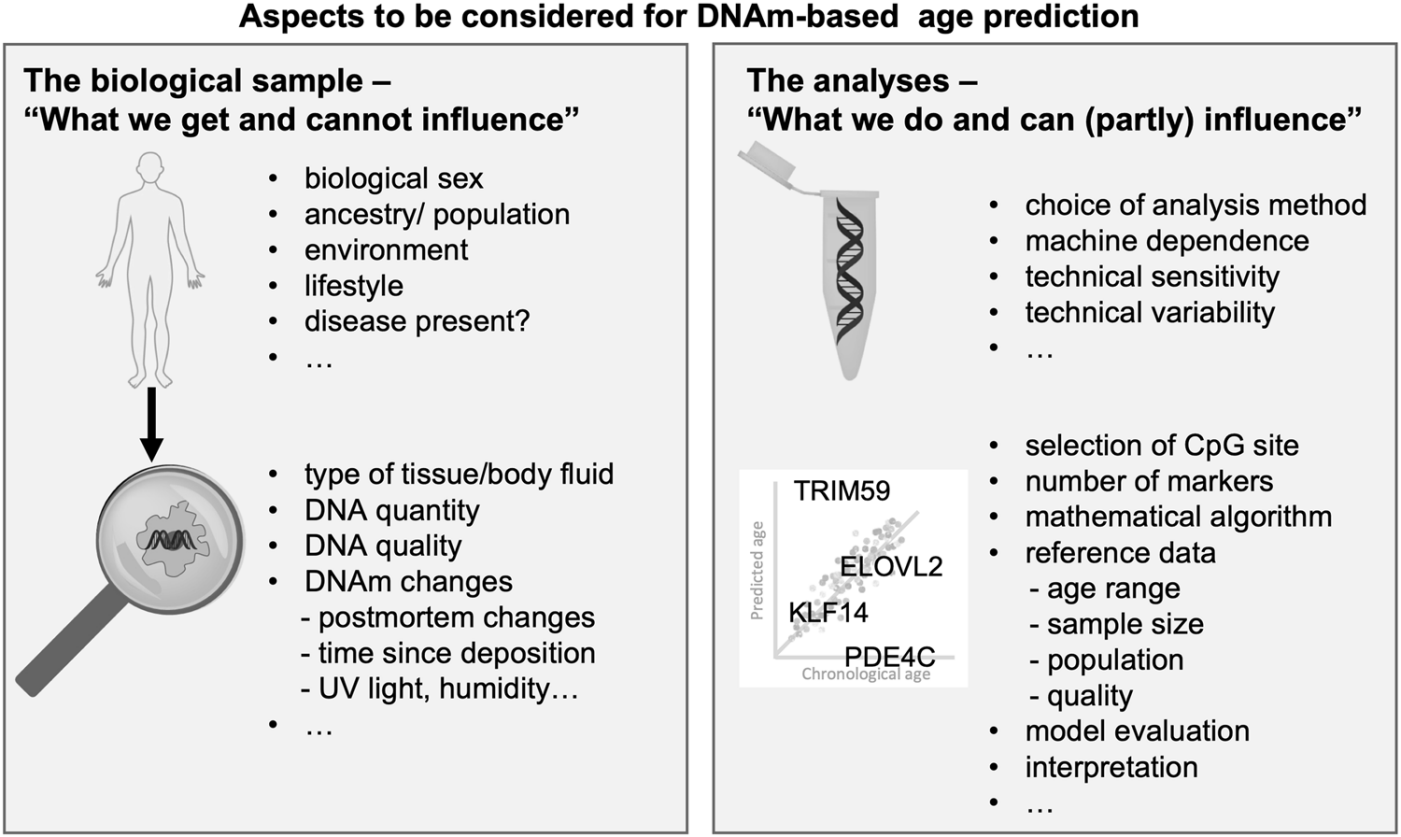
Overview of aspects to be considered in respect to the available material, DNAm analysis, and development of age prediction models. In contrast to the trace material that cannot be altered, analysis and model development are tools in which aspects can be considered and optimizations strategies applied

Within the last years, insights into the fascinating field of epigenetics increased in an expanse, which have also aroused attention in the field of forensic genetics. Until now, the use of epigenetically coded information of a trace found at a crime scene has not yet become a standard method in forensic casework laboratories. However, recent research demonstrates growing interest, and laboratories have started the development of assays for DNA methylation (DNAm) analysis, especially for tissue and body fluid identification (reviewed in An et al. 2012; Kader et al. 2020; Sijen and Harbison 2021). Additionally, research on the role of genomic imprinting for the determination of parent-of-origin alleles (Li et al. 1993; Zhao et al. 2005; Nakayashiki et al. 2009) or attempts for the authentication of DNA as biological material (Frumkin et al. 2010) was performed. Excellent reviews are available on the general usage of DNAm for forensic casework (Gršković et al. 2013; Vidaki et al. 2013; Gunn et al. 2014; Kader and Ghai 2015). The prediction of the chronological age of an individual became an intensely studied application using DNAm analysis. Many studies on age-dependent DNAm changes were often initiated by medical interest in the process of aging, including creation of epigenetic clocks (Teschendorff et al. 2010; Alisch et al. 2012; Horvath 2013; Hannum et al. 2013; Marioni et al. 2015). However, forensic scientists follow another goal, since prediction of chronological age is in focus compared to biological age and mortality risk. Furthermore, they deal with different types of challenging samples (e.g., low DNA quantity and quality), reproducibility and accuracy, as well as legal restrictions. The aim of the review is to provide an overview of concepts and considerations around markers, methods, models, and additional aspects for age prediction in the forensic setting due to available material to be analyzed (Fig. 1). The emphasis is also placed on the biological background, as age-dependent changes exist within the complex and dynamic framework of epigenetics.

## Current methods and role of age prediction for forensic purposes

Forensic age prediction is a topic with long tradition and has been applied to narrow down the age of dead individuals to assist the identification of unidentified bodies (Ritz-Timme et al. 2000). Furthermore, age verification of living individuals is important for differentiation between legal age thresholds, playing, e.g., a role in case of immigration and in court (Schmeling et al. 2004, 2006). So far, age prediction is mainly based on the analysis of morphological and physiological characteristics, which are described and discussed elsewhere (Ritz-Timme et al. 2000; Schmeling et al. 2007). Alternatives based on molecular markers have been under investigation for a long time. These can be protein changes, i.e., accumulation of racemized aspartic acid and advanced glycation products (Brownlee 1995; Ritz-Timme and Collins 2002) or nucleic acid alterations, such as a 4977-bp mtDNA deletion (Lee et al. 1994), shortening of the telomere length (Tsuji et al. 2002; Takasaki et al. 2003; Karlsson et al. 2008), signal-joint T cell receptor excision circles (sjTRECs) (Zubakov et al. 2010; Ou et al. 2012; Cho et al. 2014), as well as changes in RNA expression (Peters et al. 2015). Age dependence for most of these markers is long known, however with limited potential for application due to low accuracy or dependence on specific tissues (Meissner and Ritz-Timme 2010). The potential of RNA as a biomarker for age prediction was demonstrated (Peters et al. 2015; Fleischer et al. 2018; Ren and Kuan 2020) but a possible application in a forensic setting needs to be further clarified.

## Fundamentals of DNA methylation

For many years, the focus of forensic DNA analysis was restricted to the investigation of the ‘raw’ DNA sequence itself, primarily to determine the individual DNA profile using short tandem repeat (STR) analysis (Jeffreys et al. 1985b, a; Ellegren 2004; Jobling and Gill 2004). In addition to (almost) the same DNA sequence in all nucleated cells of a living organism, specific factors regulate our genome and thereby enable both cell-type-specific behavior and adaptations to internal as well as external influences (Feinberg 2001; Bjornsson et al. 2004; Boland et al. 2014). This concept was already proposed in 1942 by Waddington, shaping the term ‘epigenetics’ (reprint Waddington 2012). DNA methylation was proposed very early as a key factor in epigenetic regulation, first solely as an inhibitory regulator of gene expression (Riggs 1975; Holliday and Pugh 1975). However, today it is clear that regulation strongly depends on the location of methylation and thus can act both repressive and activating (Jones 2012). Other important representatives are packing structures (histones), regulatory DNA elements (e.g., enhancers), and noncoding RNAs (Goldberg et al. 2007).

DNAm (-CH3) in mammals occurs primarily at the fifth carbon atom of the base cytosine in the cytosine-guanine (CpG) sequence context. In addition, non-CpG dependent DNAm occurs but is restricted to neural and pluripotent cell types (Ziller et al. 2011; Arand et al. 2012). Hydroxymethylation (DNAhm) is a further modification regulating maintenance and differentiation of embryonic stem cells and present as an intermediate product during active removal of the methylation, respectively (Wallace et al. 2010; Hill et al. 2014; Zampieri et al. 2015).

Due to the double-stranded nature of DNA, DNAm occurs on both strands as 5′-CpG-3′ is also present on the opposite strand. Changes in the DNAm pattern occur mainly during development and cell differentiation, but are largely preserved in later cell divisions (Chen and Riggs 2011). In total, approximately 5% of cytosines are methylated (5mC) in the genome (referring to 80% of CpG positions being methylated). However, methylation is not evenly distributed throughout the genome with cell type and tissue-specific differences (Ehrlich et al. 1982; Ziller et al. 2013). In particular, long CpG-rich stretches, so-called CpG islands, contain a higher density of CpG sites (> 50%) and are mainly nonmethylated. Most of these regions (around 60%) are associated with promoters and nonmethylated sites are correlated to allow expression of the corresponding genes (Zampieri et al. 2015). In these regions, DNAm can block gene expression (Bird 1986; Cross and Bird 1995; Jones 2012), while DNAm in gene bodies can have the opposite effect (Razin and Riggs 1980; Jones 1999; Rauch et al. 2009; Lister et al. 2009; Laurent et al. 2010; Chen and Riggs 2011).

To what extent DNAm itself plays the decisive regulatory role or whether it mainly (permanently) stabilizes the epigenetic status given by histone modifications depends on the genomic location, the cell type, and time point (Jones 1999, 2012). Whereas the cell-type-specific DNAm patterns must be stable to preserve cell identity, a flexible change in DNAm can be generated by active methylation and demethylation. The latter can occur passively by loss of methylation or actively enzymatically, in particular via TET enzyme-based oxidation (Jones and Taylor 1980; Mayer et al. 2000; Oswald et al. 2000; Ma et al. 2009; Tahiliani et al. 2009; Wu and Zhang 2017). Through adaptations of DNAm to intrinsic and extrinsic changes, a stable and, at the same time, flexible chemical regulation is possible. Twin studies demonstrate that even if the genomic background is the same, epigenetic differences can be measured, caused by general epigenetic drift, as well as environmental differences (Fraga et al. 2005; Poulsen et al. 2007; Teschendorff et al. 2013; Issa 2014). Consistent with the dynamic side of DNAm, it has been shown that DNAm at some CpG positions changes in an age-dependent manner (Christensen et al. 2009; Teschendorff et al. 2010; Hannum et al. 2013).

## Aging and age-dependent DNAm changes

Aging is a universal process that is at least partially controlled by genetic pathways and biochemical processes. During aging, physiological integrity decreases, leading to impaired functioning and thereby to an increased morbidity and mortality rate (Cevenini et al. 2008; López-Otín et al. 2013). Furthermore, there are individual and environmental differences (Melis et al. 2013). López-Otín et al. defined nine 'hallmarks of aging’ of which one is epigenetic alteration in addition to altered intercellular communication, stem cell exhaustion, cellular senescence, mitochondrial dysfunction, deregulated nutrient sensing, loss of proteostasis, telomere attrition, and genomic instability. Even if these hallmarks are labeled separately, they are interconnected, affecting each other, and therefore contributing together to the aging process and the resulting phenotype (López-Otín et al. 2013). Although we all undergo this process over time, large differences between individuals are observed in the phenotype of aging. That leads to differences in the biological ages between individuals who actually have the same chronological age (the period since birth). Multiple factors such as genetic background, environment, life style, and stochastic factors can be responsible for the observed differences (Candore et al. 2006).

The first indication of age-dependent changes in DNAm was found in the 1990s, revealing that altered and de novo DNAm can be observed in the promotor of *IGF2* and the estrogen receptor in colon cells of aging individuals, as well as during cancerogenesis (Issa et al. 1994, 1996). In general, global hypomethylation is associated with age (Wilson and Jones 1983; Ca 1993; Bollati et al. 2009); however, local hypermethylation in CpG rich regions was identified (Maegawa et al. 2010; Rakyan et al. 2010; Bell et al. 2012). Genome-wide studies revealed high numbers of single age-dependent DNAm positions, of which some studies applied these to create mathematical models for age prediction (Fraga et al. 2005; Teschendorff et al. 2010; Koch and Wagner 2011; Horvath 2013; Hannum et al. 2013). These developments led to the additional terms 'epigenetic age' and 'epigenetic clocks', which refer to the measurement of a biological epigenetic marker (e.g., DNAm) and can contain information on the acceleration or deceleration of age in an individual by the difference between the measured epigenetic age and the chronological age (Horvath and Raj 2018). Some clocks were developed to explicitly predict biological age, including prediction of all-cause mortality (‘PhenoAge’, ‘GrimAge’, ‘DNAmFitAge’) (Levine et al. 2018; Lu et al. 2019; McGreevy et al. 2023). On the contrary, to create the chronological age prediction, markers have to be especially chosen based on their robustness to environmental factors, diseases, and phenotypes of an individual. Therefore, a good marker for chronological clock may not be a good candidate for biological age prediction, as good candidates were removed due to their high variation (correlated to biological variation) between individuals of the same age (Field et al. 2018). 'Robust' in this context does not mean that all possible altering conditions can be excluded, but that a prediction as robust as possible can be performed due to marker choice and use of a broad reference population trying to resemble the overall population (without selection for specific aspects such as smoking, nutrition behavior, and fitness). Therefore, division into ‘chronological epigenetic clocks’—also named ‘forensic age clock’—and ‘biological epigenetic clocks’ was proposed (Bell et al. 2019).

## Ingredients and components to predict the chronological age

The idea behind forensic age prediction is to predict chronological age with the highest possible precision. First, an analysis method must be chosen that allows robust and reliable DNAm quantification, and can be applied in a forensic laboratory (‘DNA methylation analysis methods’). Second, age-dependent DNAm markers must be identified (‘DNAm markers’). Third, mathematical models must be created using a training set that covers a broad age range based on a large number of individuals (outbalancing a diverse spectrum of environment, diseases, etc.) and evaluated with test data not included in model development (‘Basics of age prediction models’). Furthermore, special aspects such as the tissue or body fluid type (‘Models developed for different tissues and body fluids’), amount of available DNA (‘Consideration of DNA amount’), and influences on DNAm (e.g., lifestyle, disease) (‘Other potential influences on the accuracy of age prediction’) should be considered with care.

## DNA methylation analysis methods

DNAm does not change the DNA sequence itself. Therefore, it is not directly measurable via PCR and sequencing using standard approaches of forensic applications. During the PCR reaction, methylated cytosine is replaced with classical cytosine included in the PCR reaction mix, resulting in a loss of the DNAm pattern. The choice of the appropriate tool often depends not only on the task (e.g., qualitative versus quantitative analysis) but also on the amount of DNA, the optimization steps for the assay setup, the availability of analysis machines, and the costs, time, and expertise needed for processing of the samples. Therefore, only some methods will be highlighted here with an emphasis on methods used mainly in forensic epigenetics.

Three main categories of DNA pretreatment can be distinguished: (i) fixation of the DNAm pattern by bisulfite conversion; (ii) digestion of nonmethylated DNA by methylation sensitive DNA restriction enzymes (Bestor et al. 1984; Bickle and Krüger 1993; Huang et al. 1999); and (iii) selection of methylated DNA with the help of antibodies (MeDIP) (Weber et al. 2005). Although not largely present, it should be noted that these standard methods for DNAm detection cannot differentiate between DNAm and DNAhm, and the measured DNAm therefore contains the actual DNAm and the (rare) content of DNAhm.

Only bisulfite conversion will be explained in depth, as is the current gold standard for single base resolution of DNAm, and is the basis for the commonly used age prediction tools, for further reading about the other methods it is referred to (Harrison and Parle-McDermott 2011). Treatment with sodium bisulfite (sodium hydrogen sulfite) leads to sulfonation in pyrimidines (Hayatsu et al. 1970; Shapiro et al. 1973; Kai et al. 1974; Hayatsu 1976), which occurs much more slowly for methylated cytosines (Wang et al. 1980). Subsequent hydrolytic deamination and renewed desulfonation results in the formation of uracil at the positions of the originally nonmethylated cytosines. This chemical process leads depending on the kit used to some extent to DNA degradation and DNA loss (Holmes et al. 2014; Hong and Shin 2021). Bisulfite sequencing uses this approach in combination with the PCR and sequencing during which uracil is replaced by thymine (Frommer et al. 1992). Knowing by the reference sequence which position initially contained a cytosine, the DNAm status can be calculated by taking the amount of cytosine (initially methylated cytosines) divided by the amount of cytosine plus thymine (to uracil-converted nonmethylated cytosines) at that position. For analysis of the reverse strand, guanine and adenine must be considered for the calculation. For sequencing, common methods such as Sanger, massive parallel sequencing (MPS), pyrosequencing, and minisequencing (SNaPshot™) can be used. Especially pyrosequencing and MPS allow an exact quantification, as well as detection of multiple CpG sites for DNAm analysis and non-CpG sites for evaluation of the bisulfite conversion efficiency of the amplified fragments. Other possible methods also applied for DNAm-based age prediction are real-time PCR specific to methylation (Kondo et al. 2021), real-time PCR with high resolution melting (HRM) (Hamano et al. 2016, 2017), and digital droplet (ddPCR) (Shi et al. 2018; Han et al. 2020). In addition, the potential for DNAm analysis of nanopore sequencing was shown (Rand et al. 2017; Simpson et al. 2017). However, either they do not allow for the needed multiplex capacity (restricted by color channels in the case of real-time machines), enough resolution for an accurate single-based quantification, especially if multiple CpG sites are present in the amplicon (HRM), or they need specific equipment currently less used in forensic laboratories (ddPCR, Nanopore). Although not considered as a standard tool for forensic analysis, the Illumina Infinium microarray platform should not be neglected, as most of the markers today used were obtained using microarray data and the CpG ID numbering system (cg identifier) of Infinium microarrays. Three array types can be divided, 27 K, 450 K, and the EPIC version that covers more than 850,000 CpG sites (Bibikova et al. 2009, 2011; Pidsley et al. 2016). Since most of the data sets available online in recent years were based on the 450 K (and to a lesser extent the 27 K), these were often the basis for the selection of age-dependent DNAm markers (cf. ‘DNAm Markers’).

The choice of method also depends on the application. Currently, the two most important applications in forensic epigenetics are the differentiation between body fluids or tissues and the prediction of age. Although both are based on the measurement of DNAm patterns, the methodology used for age prediction must be more accurate, as changes of 1% of DNAm can be important (e. g., the mean increase in DNAm of the strong-changing marker *ELOVL2* is less than 1% per year in middle-aged individuals (Naue et al. 2017)). In particular, to examine the variation obtained by the analysis methods, some studies have examined the differences that arise due to the technology used, and others have examined whether a reliable analysis is performed by different laboratories using the same technology (Freire-Aradas et al. 2020; Holländer et al. 2021; Naue et al. 2021a). Freire-Aradas et al. analyzed 84 blood samples with Epityper, pyrosequencing, MiSeq and minisequencing, gaining comparable results with the exception of minisequencing. Using a model based on all data from the four technologies, the highest discrepancies were identified for *MIR29B2CHG* (Freire-Aradas et al. 2020). Different studies have proposed approaches to account for such variation across technologies, such as including a variable that considers the used technology (Hong et al. 2019), applying a Z-score transformation (Feng et al. 2018; Freire-Aradas et al. 2020), or building specific models for each technology (Schwender et al. 2021).

Additionally, machine-type-specific differences can occur, as observed for minisequencing on the 3130 Genetic Analyzer and the newer 3500 model in collaborative exercises performed during the last years (Holländer et al. 2021; Naue et al. 2021a; Lee et al. 2022). So and Lee performed a deeper investigation, reanalyzing samples on the 3500 originally measured with the 3130 and concluded that the original age prediction model cannot be used and a new model was created for the 3500 (So and Lee 2021). Other studies also observed differences between the initial published results and their own implementations (Daunay et al. 2019; Pfeifer et al. 2020). Taken together, these results show the importance of solid verification during the implementation of published models in the own laboratory.

## DNAm markers

The definition of ‘marker’ differs between publications and refers in the first place often to the genetic loci/sequence region and in the final model to the specific CpG position(s) analyzed. It has to be considered that a flexible biological marker (i.e., DNAm) is measured and that intra- and interindividual differences will occur even if especially markers for the purpose of chronological age prediction are selected. In the past, a large number of markers were identified in various studies based mainly on the determination of the Pearson’s product-moment correlation (r) and Spearman rank correlation (rho), respectively. Some of these markers are represented in multiple models, and were independently identified in studies generating own or using publicly available microarray data, or directly selected as potential candidates from the previous literature. Many markers were identified according to the selection criteria. Table 1 lists common markers incorporated in a final mathematical model for forensic application, at least used by two studies from different laboratories. Furthermore, markers applicable for age prediction in semen samples were also included if only mentioned once, (but partly validated in interlaboratory validation studies). However, markers were not included in the table when no model or only preliminary models without further evaluation were created due to the sample number, as, for example (Alsaleh et al. 2017; Naue et al. 2018b, 2021b; Lee et al. 2020). For the biological function of genes, it is referred to the NIH database (NCBI: https://www.ncbi.nlm.nih.gov/gene).

**Table 1:** Common age-dependent DNAm-markers used for age prediction models in previous studies

Loci for age prediction	Full gene name	DNAm change with age	Material investigated in the studies	References
ARHGEF17	Rho guanine nucleotide exchange factor 17	Down	Semen	Pisarek et al. (2021); Heidegger et al. (2022)
ASPA	Aspartoacylase	Up	Blood, bone, buccal swab teeth	Weidner et al. (2014); Bekaert et al. (2015b); Freire-Aradas et al. (2016, 2022); Pan et al. (2020); Han et al. (2020); Woźniak et al. (2021)
CCDC102B	Coiled-coil domain containing 102B	Down	Blood	Florath et al. (2014); Park et al. (2016); Freire-Aradas et al. (2016), p., (2022); Feng et al. (2018); Pan et al. (2020); Han et al. (2020, 2022); Lemesh et al. (2021); Aliferi et al. (2022)
cg12837463/ LOC401324	–	Down	Semen	Lee et al. (2015, 2018); Li et al. (2020a); Pisarek et al. (2021); Lemesh et al. (2021); Heidegger et al. (2022)
CNGA3	Cyclic nucleotide-gated cation channel alpha-3	Up	Buccal swab, saliva	Hong et al. (2017); Lemesh et al. (2021)
DDO	D-aspartate oxidase	Down	Blood	Naue et al. (2017); Shi et al. (2018)
EDARADD	Edar associated death domain	Down	Blood, buccal Swab, saliva, teeth	Bekaert et al. (2015b); Correia Dias et al. (2020b); Woźniak et al. (2021); Schwender et al. (2021); Aliferi et al. (2022); Ambroa-Conde et al. (2022)
ELOVL2	ELOVL fatty acid elongase 2	Up	Blood, bone, buccal swab, hair, saliva, teeth	Florath et al. (2014); Zbieć-Piekarska et al. (2015a); Bekaert et al. (2015b); Hamano et al. (2016); Park et al. (2016); Freire-Aradas et al. (2016, 2022); Naue et al. (2017); Feng et al. (2018); Jung et al. (2019, p. 2); Han et al. (2020, 2022); Correia Dias et al. (2020b); Hao et al. (2021); Kondo et al. (2021); Zapico et al. (2021); Woźniak et al. (2021); Lemesh et al. (2021); Aliferi et al. (2022); Ambroa-Conde et al. (2022)
EXOC3	Exocyst complex component 3	Down	Semen	Pisarek et al. (2021); Heidegger et al. (2022)
FHL2	Four and a half LIM domains 2	Up	Blood, buccal swab, saliva, teeth	Florath et al. (2014); Zbieć-Piekarska et al. (2015a); Hamano et al. (2016); Freire-Aradas et al. (2016, 2022); Jung et al. (2019, p. 2); Pan et al. (2020); Han et al. (2020, 2022); Correia Dias et al. (2020b); Zapico et al. (2021); Woźniak et al. (2021); Lemesh et al. (2021); Aliferi et al. (2022)
GALR2	Galanin receptor 2	Down	Semen	Pisarek et al. (2021); Heidegger et al. (2022)
GRIA2	Glutamate receptor ionotropic AMPA 2	Up	Blood, hair	Soares Bispo Santos Silva et al. (2015); Mawlood et al. (2016); Hao et al. (2021)
HOXC4	Homeobox C4	Up	Blood, buccal swab, saliva	Naue et al. (2017); Ambroa-Conde et al. (2022)
IFITM2	Interferon induced transmembrane protein 2	Down	Semen	Pisarek et al. (2021); Heidegger et al. (2022)
ITGA2B	Integrin subunit alpha 2b	Down	Blood, buccal swab	Weidner et al. (2014); Huang et al. (2015); Pan et al. (2020); Han et al. (2020)
KLF14	Kruppel-like factor 14	Up	Blood, bone, buccal swab, hair, saliva, teeth	Zbieć-Piekarska et al. (2015a); Vidaki et al. (2017); Alghanim et al. (2017); Naue et al. (2017); Aliferi et al. (2018); Jung et al. (2019, p. 2); Pan et al. (2020); Hao et al. (2021); Zapico et al. (2021); Woźniak et al. (2021); Schwender et al. (2021); Han et al. (2022)
LAG3	Lymphocyte activating 3	Down	Hair	Hao et al. (2021)
LDB2	LIM domain binding 2	Down	Blood	Naue et al. (2017); Lemesh et al. (2021); Aliferi et al. (2022)
MEIS1–AS3	MEIS1 antisense RNA 3	Down	Blood	Naue et al. (2017); Han et al. (2020); Lemesh et al. (2021)
MIR29B2CHG (C1orf132)	MIR29B2 and MIR29C host gene	Down	Blood, buccal swab, saliva	Zbieć-Piekarska et al. (2015a); Freire-Aradas et al. (2016, 2022); Feng et al. (2018); Jung et al. (2019, p. 2); Hao et al. (2021); Woźniak et al. (2021); Lemesh et al. (2021); Aliferi et al. (2022); Ambroa-Conde et al. (2022); Han et al. (2022)
NOX4/ FOLH1B1	NADPH oxidase 4	Up	Semen	Lee et al. (2015, 2018); Li et al. (2017, 2020a); Pisarek et al. (2021); Lemesh et al. (2021); Heidegger et al. (2022)
NPTX2	Neuronal pentraxin II	Up	Blood, saliva, teeth	Bocklandt et al. (2011); Huang et al. (2015); Mawlood et al. (2016); Zapico et al. (2021)
OTUD7A	OTU domain containing 7	Up	Blood, buccal swab, saliva	Florath et al. (2014); Ambroa-Conde et al. (2022)
PALM	Paralemmin	Down	Semen	Pisarek et al. (2021); Heidegger et al. (2022)
PDE4C	Phosphodiesterase 4C	Up	Blood, bone, buccal swab, hair saliva, teeth	Weidner et al. (2014); Bekaert et al. (2015b); Freire-Aradas et al. (2016, 2022); Feng et al. (2018); Han et al. (2020); Correia Dias et al. (2020b); Hao et al. (2021); Woźniak et al. (2021); Schwender et al. (2021); Ambroa-Conde et al. (2022)
PPP2R2C	Protein phosphatase 2 regulatory subunit Bgamma	Down	Semen	Pisarek et al. (2021); Heidegger et al. (2022)
RASSF5	Ras association domain family member 5	Up	Blood	Vidaki et al. (2017); Feng et al. (2018); Lemesh et al. (2021); Aliferi et al. (2022)
RPA2	Replication protein A2	Up	Blood	Naue et al. (2017); Lemesh et al. (2021)
SCGN	Secretagogin	Up	Blood, hair, saliva, teeth	Vidaki et al. (2017); Alghanim et al. (2017); Aliferi et al. (2018); Hao et al. (2021); Zapico et al. (2021)
SH2B2	SH2B adaptor protein 2	Down	Semen	Pisarek et al. (2021); Heidegger et al. (2022)
SLC12A5	Solute carrier family 12 member 5	Up	Blood, buccal swab, hair, saliva	Florath et al. (2014); Hong et al. (2017); Hao et al. (2021); Lemesh et al. (2021)
SST	Somatostatin	Up	Buccal swab	Hong et al. (2017); Lemesh et al. (2021)
SYT7	Synaptotagmin 7	Down	Semen	Pisarek et al. (2021); Heidegger et al. (2022)
TBX4	T-Box transcription factor 4	Down	Semen	Pisarek et al. (2021); Heidegger et al. (2022)
TRIM59	Tripartite motif con-taining 59	Up	Blood, buccal swab, saliva	Zbieć-Piekarska et al. (2015a); Naue et al. (2017); Feng et al. (2018); Jung et al. (2019, p. 2); Woźniak et al. (2021); Han et al. (2022)
TSSK6	Testis specific serine kinase 6,	Up	Buccal swab, saliva	Hong et al. (2017); Lemesh et al. (2021)
TTC7B	Tetratricopeptide repeat domain 7B	Down	Semen	Lee et al. (2015, 2018); Pisarek et al. (2021); Lemesh et al. (2021); Heidegger et al. (2022)
TUBB3	Tubulin beta 3 class III	Down	Semen	Pisarek et al. (2021); Heidegger et al. (2022)
ZNF423	Zinc finger protein 423	Down	Blood	Park et al. (2016); Pan et al. (2020)
ZYG11A	Zyg-11 family mem-ber A	Up	Blood	Florath et al. (2014); Naue et al. (2017)

One of the first studies was conducted by Bocklandt et al. in 2011 selecting a small number of CpG positions applicable for forensic use. Initially, 88 age-dependent sites were considered in the study and narrowed to three loci (*TOM1L1*, *EDARADD,* and *NPTX2*), of which the final model included *EDARADD* and *NPTX2* for the prediction of age in saliva (Bocklandt et al. 2011). The shortly after published Horvath clock included 353 CpG sites (Horvath 2013). When comparing the markers in Table 1 and the 353 CpG sites of the Horvath clock, only seven genes overlap with the loci currently used in forensic assays (*KLF14*, *ITGA2B*, *LAG3*, *NOX4*, *PDE4C*, *RASSF5*, and *SCGN*).

The probably best known and commonly used marker is *ELOVL2*, first mentioned in the publication by Garagnani et al. together with *PENK* and *FHL2* (Garagnani et al. 2012). The fact that *ELOVL2* does not overlap with Horvath’s clock is probably due to the lack of coverage of the corresponding CpG sites on the Illumina Infinium 27 K platform. Although the study also included 450 K data, only overlapping markers on both platforms were considered for the age prediction model (Horvath 2013). Multiple CpG sites in *ELOVL2* correlate (nonlinear) with age over a wide range of tissues (cf. Table 1). However, tissue-specific characteristics, such as the amount of change per year and tissue-specific DNAm shift ('baseline DNAm') exist (Slieker et al. 2018; Naue et al. 2018b). Dependent on the study, only the CpG site that was most closely related or multiple highly correlating CpG sites were integrated into the published model of a study. Some markers were used in only a few studies (e.g., *ARHGAP22*, *CNTNAP2*, cg07082267, cg26947034, *GRM2*, *NKIRAS2*, *F5*, *SYNE4*), which may be due to multiple reasons. During marker selection, the question of the most suitable marker arises. Multiple thoughts have to be considered: (1) the choice of the correlation parameter, and threshold used for selection; (2) the number of markers included, depending on the analysis method and the model algorithm; (3) the amount of DNAm increase/decrease with age to facilitate differentiation also between low age ranges and to be higher than technical noise seems favorable; (4) the purpose of having a tissue-specific model or a cross-tissue model (e.g., *ELOVL2* usable for age prediction based on multiple biological sources); (5) a marker as stable as possible also in case of disease or lifestyle/environment conditions. Furthermore, age-dependence of some markers is not always reproduced: *ITGA2B* showed only a weak correlation in blood (Bekaert et al. 2015b), but was identified before in other studies (Alisch et al. 2012; Weidner et al. 2014). Various issues such as the different age ranges covered, the tissue analyzed, the model used, and technical bias may be responsible for the observed differences. Furthermore, although the loci overlap between various studies, different CpG sites might have been used in the final model, as neighboring sites often correlate, leading to close Spearman correlation values, but might be slightly different between the studies. Furthermore, marker selection should not be handled too rigidly, as some markers might show a weaker correlation but could be useful for the reduction of outliers in a model, others might have a lower change with age, but show a very strong correlation with age (such as *KLF14*), and others might be very informative in specific tissues (such as semen). Therefore, a final assessment of the usefulness of a marker is difficult to do, and depends on the final aim (e.g., model specific for young age groups) and implementation (e.g., analysis method, mathematical algorithm).

Most research is conducted on autosomal gene regions but also the Y and X chromosome contain age-dependent CpG sites (Lund et al. 2020; Li et al. 2020b; Vidaki et al. 2021; Kananen and Marttila 2021; Jiang et al. 2023). Interestingly, different amounts of age-dependent sites were identified on the X-chromosome dependent on sex, with 1327 sites in men and only 325 sites in women, of which 122 CpG sites overlapped and five additional sites showed opposite age-dependent directions (Kananen and Marttila 2021). Especially, Y-chromosome-based age prediction would have advantages for forensic purposes, being male-specific and therefore usable in male–female DNA mixtures for age prediction of the male contributor. Lund et al. found between 40 and 169 Y chromosome CpG sites within four examined datasets, of which at least 82% of CpG sites showed hypermethylation, including seven CpG sites that overlapped the datasets (Lund et al. 2020). On the contrary, Kananen and Marttila found 46 age-dependent sites in at least two of the five analyzed datasets, but only two CpG sites overlapped in four of them. However, these two CpG sites did not overlap with the seven sites of Lund et al. That might be due to different age ranges, selection criteria, and technical differences (noise) between the data sets.

An additional interesting target for forensics would be DNAm in mtDNA (mtDNAm), as it would be especially useful in degraded samples that lack enough nuclear DNA. Controversial results were published, as different regions were analyzed, various methods applied for the analysis and the limited DNA conversion efficiency of circular DNA led to an overestimation of mtDNAm in some studies (Liu et al. 2016). A review by Cao et al. summarizes the observed difficulties and concludes that mtDNAm is on average between 1.5 and 5%, with some non-CpG sites reaching 10%, and has an asymmetric behavior (as the L-strand has a higher C-content) (Cao et al. 2021). Two recent studies detected age-dependent differences in postmortem brain tissue, with a low increase with age (< 10% DNAm), confirming the general low level of mtDNAm (Huang et al. 2022; Devall et al. 2023). The overall picture remains difficult, and more research is needed to get a deeper understanding of the potential of mtDNAm.

## Basics of age prediction models

The selection of DNAm markers described above is the first step in model development. Age prediction models are trained using reference data (measured DNAm data and chronological age). The included DNAm markers are the initially selected features. Until now, a large number of age prediction models have been created. Most research groups created their age prediction model using one pre-selected algorithm, for example, multivariate linear regression (MLR) (Woźniak et al. 2021), multivariate quantile regression (Ambroa-Conde et al. 2022), random forest regression (Naue et al. 2017), and artificial neural networks (Vidaki et al. 2017). In other studies, multiple models were initially tested to select the best (Xu et al. 2015; Smeers et al. 2018; Aliferi et al. 2018; Freire-Aradas et al. 2022; Yang et al. 2023).

Independent of the model used, it is important that a training data set is used for model development and that independent data (not involved in model development) are used for the evaluation (Alzubi et al. 2018). In case of a low sample number, cross-validation (CV) methods (k-fold CV, leave-one-out (LOOCV)) can be a useful alternative. However, the use of an independent dataset, with an independent preparation of the samples, is advantageous for evaluating additionally intralaboratory batch effects between experiments. Interlaboratory exercises and validations would additionally allow the evaluation of batch effects between laboratories, as done in (Holländer et al. 2021; Naue et al. 2021a; Lee et al. 2022). During modeling, various things have to be considered. It is important to avoid an overfitted model, which can happen if the model parameters are chosen to perfect fit the training data set but are set too stringent for analysis of independent test data. This risk can be minimized by an initial feature selection using completely different data. Multiple studies realized this by using publicly available microarray data for initial marker selection, (among others Bocklandt et al. 2011; Weidner et al. 2014; Vidaki et al. 2017; Naue et al. 2017; Freire-Aradas et al. 2022).

Model evaluation is performed primarily using the mean absolute error (MAE), or the root mean square error (RMSE) (Handelman et al. 2019). Furthermore, the median absolute evaluation can be used, which is also sometimes abbreviated MAE, and should not be mixed with the mean absolute error when comparing models (therefore, abbreviated MedAE in this review). Additionally, the percentage of correct predictions within in an acceptable error range (mainly ± 5 years) is often stated as in (Zbieć-Piekarska et al. 2015a; Pan et al. 2020; Freire-Aradas et al. 2022). All these values are useful for evaluating the overall model; however, they do not provide information about the maximum observed deviation between predicted and actual age, nor about the confidence in a single prediction.

Comparisons between models based on their MAE or RMSE should be made with caution. An increased range between DNAm values from individuals of the same age was measured especially in the elderly (Fraga et al. 2005; Martino et al. 2013) and was also measurable by the increasing MAEs in age group-specific analyses (Bekaert et al. 2015b; Naue et al. 2017). Therefore, a model that covers a wide age range, and is tested in many older individuals can result in a worse overall MAE compared to a model tested with a larger dataset of young individuals. Furthermore, different models are based on various needs and therefore compromise, e.g., ease of implementation in a forensic laboratory, number of analyzable markers, accurate predictions for a specific age range, tissue specificity, and need of a universal approach, respectively.

## Models developed for different tissues and body fluids

Age-dependent DNAm changes are tissue-specific and must be considered (Day et al. 2013; Slieker et al. 2018)*.* Although the Horvath epigenetic clock was created as a universal clock, it clearly shows differences in prediction accuracy between tissues with an overall MedAE of 3.6 years in the overall test data, with 3.7 years for whole blood, but 18 years for skeletal muscle (Horvath 2013). Therefore, many studies have been conducted to identify age-dependent markers specific for a tissue or to adapt the model to the reference data for each tissue. Table 1 does not claim to provide all available studies, as far more were published, and in addition yet unpublished and modified models exist, respectively. Many models developed for forensic purposes show an MAE (often referred as accuracy) of 3–5 years (cf. sections below). An overview of forensically motivated studies is provided below for analysis of different types of tissue commonly encountered in criminal investigations.

### Blood

Initially, most of the studies were developed and optimized for DNA analysis from blood (among others Weidner et al. 2014; Zbieć-Piekarska et al. 2015a, b; Huang et al. 2015; Bekaert et al. 2015a, b; Park et al. 2016; Freire-Aradas et al. 2016, 2022; Thong et al. 2017; Vidaki et al. 2017; Cho et al. 2017; Naue et al. 2017; Aliferi et al. 2018, 2022; Jung et al. 2019; Daunay et al. 2019; Alsaleh and Haddrill 2019; Han et al. 2020, 2022; Correia Dias et al. 2020b). Zbieć-Piekarska et al. developed one of the first models with an MAE of 3.9 years (Zbieć-Piekarska et al. 2015a). The set of markers analyzed (*ELOVL2*, *FHL2*, *KLF14*, *TRIM59,* and *MIR29B2CHG* (initially named *C1orf132*)) is the most investigated set. It was further evaluated applying other mathematical algorithms, using other methods and populations (Cho et al. 2017), and in studies investigating the effect of diseases (Spólnicka et al. 2016, 2018b, c). Furthermore, these markers were also independently identified and/ or implemented by other studies included in this review (Table 1).

Blood samples from deceased individuals were also examined and no generally biased DNAm results have been found so far (Hamano et al. 2016; Naue et al. 2018b; Correia Dias et al. 2020a; Pfeifer et al. 2020). However, these studies did not contain a detailed systematic investigation of the effect of different stages of putrefaction. Another possible point to consider is the cell type composition, whose role for DNAm analysis was examined in larger microarray studies and normalization procedures were developed (Houseman et al. 2012; Teschendorff et al. 2017). However, these methods require the use of microarray data for cell type deconvolution and are therefore not suitable for forensic purposes. Although the observed changes in DNAm may be correlated with changes in cell type with aging, these do not necessarily interfer with age prediction but can refer to markers highly specific to blood. Jaffe and Irizarry analyzed the association of blood cell composition and DNAm and found loci with statistically significant different DNAm depending on cell type count (including the well-known *FHL2*) (Jaffe and Irizarry 2014).

Studies using age-dependent sites on the Y chromosome have also been conducted so far only in blood. Vidaki et al. developed a support vector machine (SVM) radial model, resulting in an MAE of 7.54 years (75 CpG sites) and 8.46 years (reduced selection of 19 CpGs) for the validation set. Interestingly, in contrast to autosomal age prediction, Y-based prediction did not increase in the elderly (Vidaki et al. 2021). Very recently, Jiang et al. developed an age prediction model based on minisequencing and random forest regression (including 13 CpGs) with an MAE of 5.73 years in the test set, including individuals between 21 and 100 years (Jiang et al. 2023).

### Saliva and buccal cells

Saliva is a common trace material and the first material on which an age prediction model with an MAE of 5.2 years (LOOCV) was established for forensic purposes (Bocklandt et al. 2011). Saliva was investigated in further studies using the same markers as for blood-based models and/ or markers specific for saliva (Hong et al. 2017; Hamano et al. 2017; Jung et al. 2019; Ambroa-Conde et al. 2022). Further studies used buccal swabs as tissue source (Bekaert et al. 2015a; Pfeifer et al. 2020; Han et al. 2020; Woźniak et al. 2021; Schwender et al. 2021). Although not a trace material, buccal swabs are often used (and are easily applicable) in research studies and could be useful for age verification in living individuals.

However, buccal cell swabs and saliva cannot be considered as interchangeable material for epigenetic analysis. Saliva is a heterogeneous body fluid with a mixture of leukocytes and epithelial cells of the oral cavity. As a result, the composition of cell types may be more related to blood or to buccal swabs. However, also a buccal swab is heterogeneous, as leukocytes are also obtained during sample collection (Theda et al. 2018). To account for these heterogeneous materials, cell-type-specific markers can be included to determine the epithelial/leukocyte ratio. In one study, CpG sites in *CD6* (cg07380416) and *SERPINB5* (cg20837735) DNAm were analyzed obtaining a ‘Buccal Cell Signature’ that together with the 3-CpG age prediction set improved age prediction by decreasing the MAE from 7.03 years (3-CpG model) to 5.09 years (5-CpG model) in the independent validation set (Eipel et al. 2016). In another study, the authors were able to improve their model by analyzing a cell type-specific CpG site in *PTPN7* (cg18384097), resulting in an MAE of 3.15 years in contrast to the MAE of 4.1 years without *PTPN7* inclusion. In particular, they were able to reduce partially the deviation from chronological age in the elderly group (Hong et al. 2017). The genes *PTPN7* (coding the nonreceptor protein tyrosine phosphatase type 7) and *CD6* (a T cell differentiation gene) show hypermethylation in epithelial cells and hypomethylation in blood cells, while *SERPINB5* (serpin peptidase inhibitor clade B member 5) shows the opposite DNAm pattern with hypomethylation in epithelial cells and hypermethylation in blood (Eipel et al. 2016; Hong et al. 2017). Ambroa-Conde et al. used another approach considering the difference between saliva and buccal swab material, developing a combined minisequencing assay for buccal cells and saliva, resulting in a MedAE of 3.66 years. Furthermore, they tested the inclusion of CpG sites in *HUNK* and *RUNX1* to predict the tissue source as an additional covariable for the age prediction tool, which did not improve age prediction but led to correct classification of the tissue source in 83.69% of the cases (Ambroa-Conde et al. 2022).

Two studies were able to analyze DNAm markers of buccal swab material from deceased individuals (Naue et al. 2018b; Koop et al. 2021). Koop et al. investigated whether the decomposition stage has an influence on *PDE4C* DNAm. No dependence on the decomposition stage was found as long as enough DNA was recovered. Furthermore, no association was detected between DNA degradation until the decomposition stage six, at which tissues started to dry out. A higher amount of buccal cells and DNA was even obtained in cases of mid-level decomposition, which the authors speculate could be due to decreased mucosal stability and, therefore, easier cell collection (Koop et al. 2021).

### Semen

Unlike somatic tissues that show global hypomethylation and regional hypermethylation, in semen the opposite trend can be observed, and using the mean DNAm values of 51 regions analyzed by microarray, Jenkins et al. were able to construct an MLR model with an MAE of 2.37 for the ten independent test samples (Jenkins et al. 2018). However, only a few studies have been conducted with emphasis on forensic applications, including an exploratory study and a validation by Lee et al. in 2015 and 2018. In these studies, one CpG in *TTC7B*, cg12837463, and *NOX4,* respectively, were analyzed by minisequencing to obtain an MAE of 4.8 years (Lee et al. 2015, 2018). Li et al. also included *TTC7B* and *NOX4* in their age prediction tool using pyrosequencing and linear regression to obtain an MAE of 4.16 in fresh samples and 4.39 years in aged semen samples (Li et al. 2020a). Furthermore, the VISAGE consortium selected age-dependent sites in semen and conducted interlaboratory studies to verify the robustness of DNAm obtained from 13 markers (Heidegger et al. 2022). The final model is based on the MPS analysis of 6 CpG sites in five genomic regions (*SH2B2*, *EXOC3*, *GALR2*, *IFITM2*, *FOLH1B*) and led to an MAE of 5.1 years (RMSE 6.3 years) using an MLR model (Pisarek et al. 2021).

### Hair

Hair is often found at crime scenes, therefore two studies have investigated age-dependent changes in the hair follicles of dead or living individuals using MPS (Naue et al. 2021b) or minisequencing (Hao et al. 2021), respectively. In the case of the latter study, an MLR prediction model comprising 10 CpG sites was developed and an MAE of 4.15 years (RMSE 4.92 years) in the test set was obtained. No correlation with sex or hair color was found (Hao et al. 2021), but the plucked hair follicle can be very heterogeneous in the amount of DNA obtained, which is an important factor for successful DNAm analysis (Naue et al. 2021b). Further studies are needed and the application has its limits, as hairs found at crime scenes are mainly telegenic hairs or only hair shafts.

### Bone

So far, some studies have investigated age-dependent DNAm in bones (Shi et al. 2018; Naue et al. 2018b; Gopalan et al. 2019; Lee et al. 2020; Woźniak et al. 2021; Becker et al. 2021; Correia Dias et al. 2021). In a larger study, Woźniak et al. included, in addition to other types of tissue, 161 bone samples from the occipital bone or femoral shaft, of which 112 were used for model training and 49 for testing. Two selected sites in *ELOVL2* and *PDE4C* each, as well as one CpG site in *KLF14* and *ASPA* each*,* were analyzed by MPS and implemented in a bone-specific MLR model. The obtained MAE of 3.4 years was comparable to the results from blood with the blood-specific model developed in the same study (MAE of 3.2 years). They also tested whether their bone model could be used to predict the age of other types of tissue. Age prediction of blood samples resulted in a quite good estimate with an MAE of 4.9 years, while cartilage and muscle predictions were much less accurate with 25.8 and 13.7 years, respectively (Woźniak et al. 2021).

### Teeth

Another material with potential to predict the age of especially deceased individuals are teeth samples. Several studies have been conducted so far investigating markers also identified in other tissue types (among others *ELOVL2*, *FHL2*, *EDARADD,* and *PDE4C*). These confirmed the presence of age-dependent changes also in teeth and the potential to build models for age prediction (Bekaert et al. 2015b; Márquez-Ruiz et al. 2020; Kondo et al. 2021; Zapico et al. 2021; Correia Dias et al. 2021). Interestingly, Giuliani et al. found that the part of the tooth as a source of DNA plays an important role. Not only the amount of DNA obtained, but also the accuracies of the age predictions were different. Dental pulp material resulted in slightly better results (MedAE 2.25 years) than cementum (MedAE 2.45 years), while age prediction from dentin samples was the least accurate (7.07 years). The best result with an MedAE of 1.2 years was obtained by a combination of pulp and cementum. The authors speculate that the differences could be due to different types of dentin during life, with tertiary dentin (secreted in response to external damage) being different between individuals leading to a greater variability in age prediction, especially for the elderly (Giuliani et al. 2016). However, further evaluation is needed with additional independent samples.

## Consideration of DNA amount

There is a general need to obtain a reliable result for the analysis of trace material, including small and degraded amounts of DNA. However, in case of DNAm analysis, an additional layer of variation for the successful outcome has to be considered since the DNAm at one CpG site of one DNA molecule is a bivariate characteristic (methylated versus unmethylated). The DNAm level between 0 and 100% represents the DNAm of a cell population and, therefore, over a number of different cells. As traces often contain only a few cells of this population, different traces from the same original source can contain different DNAm patterns (Naue et al. 2018a). It should be noted that the DNAm still presents the biological value of the few analyzed cells, but not necessarily the overall tissue DNAm of an individual. Furthermore, the final measured deviation also contains technical variation. The influence of the DNAm variation on the age prediction model has to be evaluated, and might vary depending on markers, models, and the investigated age range. Therefore, multiple studies investigated the sensitivity threshold for their own assays and markers, with varying results between 1 and 20 ng (Zbieć-Piekarska et al. 2015b; Hong et al. 2017; Heidegger et al. 2020, 2022; Woźniak et al. 2021; Aliferi et al. 2022). However, these results cannot be directly compared, as the terms ‘sensitivity’, ‘robust’, and ‘reliable’ do not have a consistent definition for DNAm analysis and age prediction. Additionally, there are differences in the setup of the assay (elute volume from bisulfite conversion used for PCR, analysis methods, tissue type, and fragment sizes). Woźniak et al. were able to robustly quantify the DNAm of most markers down to 20 ng DNA input for bisulfite conversion, referring to approximately 8.8–11.8 ng converted DNA in the PCR (Woźniak et al. 2021). The same research consortium obtained stable normalized read depth and accurate DNAm results for all markers at 50 ng input for conversion and a possible 11–14.8 ng input in PCR for the analysis of semen samples (Heidegger et al. 2022). Within the same range, the results of other studies that developed an age prediction assay in buccal cells and saliva, leading to a minimum amount of 10 ng (Ambroa-Conde et al. 2022). An increased absolute error in the age prediction between duplicates or triplicates was also obtained in other studies using down to 2.5 ng and 1 ng, respectively in a pyrosequencing and MPS assay (Zbieć-Piekarska et al. 2015b; Heidegger et al. 2020)**.** However, Aliferi et al. were able to still get reproducible results down to 5 ng of DNA input for bisulfite conversion, resulting in 1 ng of converted DNA for PCR, demonstrating possible high sensitivity for age prediction (Aliferi et al. 2022). Using minisequencing, a technical limit can be the appearance of allelic dropout, resulting in a threshold of 4 ng and 5 ng of converted DNA for two assays investigating saliva and semen samples (Hong et al. 2017; Lee et al. 2018). Jiang et al. also evaluated the sensitivity for their Y-based assay. A complete electropherogram was obtained down to 0.5 ng (Jiang et al. 2023).

## Other potential influences on the accuracy of age prediction

Epigenetic modifications form a regulatory layer and can be influenced by genetics and environmental effects. The attribution of each factor is especially observable in twin studies (Fraga et al. 2005; van Dongen et al. 2016; Hannon et al. 2018; Reynolds et al. 2020). Therefore, its influence on age-dependent markers must be investigated. As many factors may contribute to the variation, only some studies can be highlighted. Although divided into sections, influences can be interconnected and may cover other smaller effects.

### Biological sex

Generally, sex-dependent differences in DNAm have been reported (Fuke et al. 2004; El-Maarri et al. 2007; Boks et al. 2009; Hannum et al. 2013; Marttila et al. 2013; Zaghlool et al. 2015). Therefore, a possible biological sex dependence on age prediction was considered since the development of the first forensic age prediction models. No significant sex-dependent differences were found in most studies developing prediction models (Koch and Wagner 2011; Bekaert et al. 2015b; Eipel et al. 2016; Freire-Aradas et al. 2016; Vidaki et al. 2017; Aliferi et al. 2022). Non-significant tendencies were observed in other studies (Zbieć-Piekarska et al. 2015a; Naue et al. 2017). Since there may be small differences depending on the markers and models chosen, future models should also be checked for differences due to sex, but, so far, it can be concluded that if sex is having an influence, then the impact on the accuracy of age prediction is rather low.

### Reference population

Furthermore, the reference population of the model has to be considered. General population-dependent DNAm differences were found by investigating genome-wide DNAm profiles of different populations, which are caused by genetic and environmental differences (Fraser et al. 2012; Heyn et al. 2013; Gopalan et al. 2017; Carja et al. 2017). Most age-dependent markers were initially identified by a combination of various publicly available data sets covering various worldwide populations (Horvath 2013; Hannum et al. 2013; Vidaki et al. 2017; Naue et al. 2017; Aliferi et al. 2022). However, the available data sets are not evenly distributed, and not all geographic regions are covered by these studies. Furthermore, many final prediction models were created on samples collected at the geographic location of the research laboratories that performed the study, e.g., residents of the Netherlands (Naue et al. 2017), the UK (Aliferi et al. 2022), South Korea (Hong et al. 2017), Poland (Zbieć-Piekarska et al. 2015a), and Germany (Schwender et al. 2021). Although not specified in detail, it can be assumed that the biogeographic ancestry of some of the individuals may be different, as well as the period of residence (and therefore environmental exposure duration) at the sampling location.

Various studies included investigations of DNAm differences between populations or validated published models for applicability in another population than the one included for model development (Eipel et al. 2016; Vidaki et al. 2017; Cho et al. 2017; Fleckhaus et al. 2017; Daunay et al. 2019; Aliferi et al. 2022). The results obtained are heterogeneous, since no differences were obtained in some studies (Eipel et al. 2016; Vidaki et al. 2017; Aliferi et al. 2022), while differences were observed in other studies (Cho et al. 2017; Gopalan et al. 2017; Fleckhaus et al. 2017; Becker et al. 2022). However, these differences were versatile. Cho et al. obtained a consistent general performance of the model analyzing South Korean individuals with a model based on individuals from Poland. However, the degree of age correlation showed differences, resulting in a retraining of the model for further improvement (Cho et al. 2017). Another study saw different amounts of interindividual variation for *ELOVL2* depending on ancestry (Fleckhaus et al. 2017), while Daunay et al. validated six existing models based on other populations for their prediction accuracy in a French population, obtaining a general lower model accuracy for some of the models (Daunay et al. 2019). So far, a generalization is not easily possible, as observed differences could be specific for the investigated population, being partly a technical and/or sample batch effect, be caused by a different setup (marker, age range, analysis method), and a combination of all factors, respectively. Another issue to consider is that the reference age is provided by the participants. Gopalan et al. accounted for the role and difficulties of age verification for specific populations by including birth and wedding certificates, school records, local and historical events, and other forms for cross-verification (Gopalan et al. 2017).

### Environmental exposures and lifestyle

The influences of various environmental exposures, such as air pollution, lead, mercury, or bisphenol A, on genome-wide DNAm changes has been observed in various studies and were summarized and discussed by Martin and Fry in 2018 (Martin and Fry 2018). Lifestyle factors such as smoking and alcohol use disorder are also often associated with changes in DNAm patterns as reviewed in (Zahs et al. 2012; Lee and Pausova 2013; Zhang and Gelernter 2017; Kaur et al. 2019; Zong et al. 2019). Regarding the forensic age prediction tools developed, Aliferi et al. found no impact on their prediction model due to environmental and lifestyle differences between the sampled individuals from the UK and Spain (Aliferi et al. 2022). Eipel et al.*,* as well as Schwender et al.*,* specifically analyzed the effect of smoking, and found no smoking-associated differences in their buccal swab samples at 5 CpG sites in *ASPA*, *ITGB2B*, *PDE4C*, *CD6* and *SERPINB5*, and 88 CpG sites in the loci of *PDE4C*, *ELOVL2*, *ITGA2B*, *ASPA*, *EDARADD*, *SST*, *KLF14*, *SLC12A5*, respectively (Eipel et al. 2016; Schwender et al. 2021). Piniewska-Róg et al. investigated DNAm changes at 44 CpG sites in *ASPA*, *EDARADD*, *ELOVL2*, *FHL2*, *KLF14*, *MIR29B2CHG*, *PDE4C*, and *TRIM59* in deceased extensive alcohol abusers. However, only an effect was observed in *MIR29B2CHG,* without a relevant impact on the age prediction using the blood-based VISAGE enhanced age model (including CpGs in *ELOVL2*, *FHL2*, *KLF14*, *MIR29B2CHG,* and *PDE4C*) (Piniewska-Róg et al. 2021).

The influence of extreme sport was also investigated as possible impact. Age predictions of elite athletes resulted in increased predicted ages, especially caused mainly by *KLF14* and *TRIM59*. The effect was more pronounced (especially due to changes in *TRIM59*) for men and women who perform power sports (Spólnicka et al. 2018a).

### Disease and medical treatment

Furthermore, DNAm changes associated with a disease can lead to changes in the precision of age prediction. Some diseases might be the result of an environmental exposure or lifestyle; thus, a changed DNAm may be the result of the lifestyle as well as the disease (cf. excessive alcohol consumption covered before). The following examples show the complex nature and possible impact of this topic.

For example, in the case of chronic lymphocytic leukemia, age in patients was not correctly predicted anymore using the markers *ELOVL2*, *MIR29B2C*, *TRIM59*, *KLF14,* and *FHL2* (Spólnicka et al. 2018c). Spólnicka et al. also investigated the effect of allogeneic hematopoietic stem cell transplantation on the prediction of recipient age. They found that the measured age of the recipient after transplantation was more correlated with the chronological age as well as the with the age prediction model calculated age of of the donor than with the chronological age of the recipient, confirming the observations of Weidner et al. (Weidner et al. 2015). However, Spólnicka et al. observed with their age prediction model a mean recipient age prediction that was 3.7 years lower than that of the donor, while the prediction in the Weidner et al. study was 7 years higher than the chronological age of the donor. The authors found the reason for the lower predicted age in hypermethylation of *MIR29BCHG2* (the higher the DNAm of *MIR29BCHG2*, the lower the predicted age), a marker that is not present in the model of Weidner et al. (Weidner et al. 2015; Spólnicka et al. 2016). Both studies had a maximum period of one year. Therefore, it might be interesting to see whether the observed effects are constant or change over a longer period. Spólnicka et al. questioned whether there is a dissociation of the age dependency of *MIR29BCHG2* and specific circumstances, and a rethinking of the usefulness of this marker for forensic age prediction would be needed (Spólnicka et al. 2016). However, they also found in another study that this marker is stable in other diseases, while *TRIM59* and *KLF14* showed hypermethylation in early-onset Alzheimer’s disease, and hypermethylation of *TRIM59* and hypomethylation of *FHL2* in Graves’ disease (Spólnicka et al. 2018b). Although single-marker-based predictions led to large discrepancies between chronological and prediction age (up to 10 years), using the original 5-marker age prediction model, an increase of only 1.7 years was observed for early-onset Alzheimer's disease in the entire age range (6 years in the younger group). No bias was found in the case of Graves’ disease, which can be explained by the opposite changes in DNAm of *TRIM59* and *FHL2* (Spólnicka et al. 2018b). Having a look at various diseases, Aliferi et al. found no bias due to schizophrenia, rheumatoid arthritis, frontal temporal dementia, and progressive supranuclear palsy for their 11-marker model based on blood. However, at the gene level, they conclude that there are potential associations with obesity, smoking, metabolic, and cardiovascular diseases. This does not directly lead to an effect on the accuracy of age prediction, but associations between age markers and key changes during aging must be considered (Aliferi et al. 2022).

These examples reinforce the advantage of using multiple markers for age prediction. The overall observed differences of the impact on age prediction are not surprising as the markers analyzed as well as the tissue source investigated will be affected differently in case of an underlying disease.

### Consequences

Information about the biological sex of a person can be determined during standard STR-profiling and could therefore be easily considered if needed. On the contrary, biogeographic ancestry analysis is currently limited to classification of continental regions of East Asia, South Asia, Europe, sub-Saharan Africa, Oceania, America (indigenous population) and is restricted due to legal restrictions in various countries (Schneider et al. 2019). Although the results have been controversial, knowledge of the biogeographical ancestry of the trace causer would still be beneficial for forensic application. The investigator could be more cautious with the interpretation of the result obtained. Nevertheless, the determination about the biogeographic ancestry does not allow conclusion about the residence location of the individual and therefore environmental exposures. Furthermore, the background on lifestyle and disease will not be known in the case of trace material (assumptions might be possible if a trace is found in connection with a specific lifestyle, for example, on a cigarette butt) and could only be considered if an age verification of a living individual is required. Common environmental exposures, as well as lifestyles such as smoking and moderate alcohol consumption will be covered in most prediction tools; as a part of the individuals included for training will also have this lifestyle. Knowledge of disease in the reference data is more complex, even if a ‘healthy control group’ was included. General predispositions, unknown diseases, and conditions related to the aging process will still be included in the assays and be present in the individual who left the analyzed trace.

## Combined use of methods for age prediction

Although this review focuses on age prediction using DNAm changes, the combination of biomarkers could be a useful approach. The use of a second estimator could verify or question the predicted age by DNAm analysis. So far, only a few studies have investigated this potential. Márquez-Ruiz et al. found no relevant improvement in age prediction accuracy when telomere length was combined with DNAm in a small study that examined teeth (Márquez-Ruiz et al. 2020). Zubakov et al. compared the potential of mRNA, sjTREC, telomere length and DNAm and confirmed that the highest precision was due to DNAm analysis, but that mRNA provided additional independent information useful for a combined analysis (Zubakov et al. 2016). Another example is the combination of DNAm and sjTREC as done by Cho et al. obtaining an improved prediction for the elderly (Cho et al. 2017). Other studies investigated the combination of different kinds of age-dependent changes such as the combination of skeletal, dental age, and DNAm in children (Shi et al. 2018) or the idea of combining age-dependent protein changes and DNAm (Becker et al. 2021). Even if only small improvements might be observed by adding an additional layer to the prediction, the identification of outliers and perhaps a better age prediction of these would also be an important improvement for forensic purposes.

## Mammalian age prediction

As aging is not restricted to the human species, age prediction can also be performed for other types of animal. Lu et al. constructed three universal pan-mammalian clocks using cytosines in highly conserved DNA stretches of 185 mammalian species (19 taxonomic orders) including 59 tissue types and an age range from prenatal to 129 years. As seen in humans, age-dependent cytosines are enriched at polycomb sites. The basic clock included all available animals without adaptions to different species conditions, whereas a normalized universal relative age clock considered the maximum lifespan, and a third clock normalized to sexual maturity and gestation time. Each clock was built from fewer than 1,000 CpG sites, and the chronological age versus the predicted age showed a median error of less than a year. However, species-specific differences occurred, and a lower correlation was achieved for example for bowhead whales (Lu et al. 2021). First age prediction clocks for specific animals with potential forensic relevance were also developed, including horses, dogs, cats, elephants, and apes (Ito et al. 2018; Prado et al. 2021; Horvath et al. 2021, 2022a, b; Raj et al. 2021).

## Conclusions and outlook

This review aimed to provide an overview of DNAm analysis for age prediction, including various aspects to consider. Often it was only possible to provide examples. The application of DNAm for age prediction is reasonable and multiple tools were developed. In the future, the focus should be on identifying the sources that lead to outliers to optimize the models by or to identify a possible prediction outlier. Multimarker models seem favorable for that, outbalancing single DNAm changes, and facilitating outlier detection. However, the number of markers should be within the range of the possibilities of multiplex PCRs, avoiding also the need for too much DNA.

Although most of the models referred to were developed for one type of tissue, some were developed to allow the analysis of multiple tissues. Different approaches are possible: (i) analysis of the same markers, but in part use and considering different CpG sites within different tissue-specific models as in the enhanced age prediction panel of the VISAGE consortium for age prediction using blood, buccal cells or bone material (Woźniak et al. 2021), (ii) measurement and use of the same CpG sites but with tissue-specific models as done by Jung et al. in the minisequencing assay for age prediction using blood, buccal cells, and saliva (Jung et al. 2019), and (iii) application of a universal model including various tissues and cell types (Horvath 2013). Although a universal model appears favorable, the accuracy of the model varied depending on the tissue type, and therefore, tissue-specific models based on one flexible experimental assay as used in (i) and (ii) currently are more promising for application in forensic investigations.

To allow for better comparability between studies in the future, the use of CpG sites present on microarrays might be favorable because they are investigated in a large number of studies. Although the best CpG site may have varied between studies, this phenomenon needs to be further investigated, as the sample size and batch effects can result in small differences in the correlation values. As various analysis methods will be used in the future, normalization strategies or methods allowing reliable machine-independent absolute quantification are needed. Furthermore, the application for trace material has not yet been fully evaluated. More studies on DNA quantification with respect to DNA quantity and quality, as well as changes caused by trace exposure to factors such as ultraviolet light and the time since deposition, must be performed. In the case of deceased individuals, more information on postmortem stability is needed for more tissues. Furthermore, not all types of trace material were investigated to the same depth, as for some only first attempts were made (e.g., menstrual blood, vaginal secretion in (Alsaleh et al. 2017)). In particular, more interlaboratory exercises will help to further optimize and implement age prediction tools in forensic laboratories (Holländer et al. 2021; Naue et al. 2021a; Heidegger et al. 2022; Lee et al. 2022).
